# Inhibition of leptin-induced vascular extracellular matrix remodelling by adiponectin

**DOI:** 10.1530/JME-14-0027

**Published:** 2014-10

**Authors:** Zhi Zhang, Fang Wang, Bing-jian Wang, Guang Chu, Qunan Cao, Bao-Gui Sun, Qiu-Yan Dai

**Affiliations:** Department of Cardiology, School of Medicine, Shanghai First People's Hospital, Shanghai Jiao Tong University, Shanghai, 200080, People's Republic of China; 1 Department of Cardiology, Huai'an First People's Hospital, Nanjing Medical University, Jiangsu Province, 223300, People's Republic of China

**Keywords:** leptin, adiponectin, extracellular matrix remodelling, 3D vessel model, hypertension, obesity

## Abstract

Vascular extracellular matrix (ECM) remodelling, which is the result of disruption in the balance of ECM synthesis and degradation, induces vessel fibrosis and thereby leads to hypertension. Leptin is known to promote tissue fibrosis, while adiponectin has recently been demonstrated to be anti-fibrogenic in tissue fibrosis. In this study, we aimed to evaluate the leptin-antagonist function of adiponectin and to further elucidate the mechanisms through which adiponectin dampens leptin signalling in vascular smooth muscle cells, thus preventing excess ECM production, in our already established 3D co-culture vessel models. Our 3D co-culture vessel model, which mimics true blood vessels, is composed of vascular endothelial cells, vascular smooth muscle cells and collagen type I. We validated the profibrogenic effects of leptin and analysed matrix metalloproteinase 2 (MMP2), MMP9, tissue inhibitor of metalloproteinase 1 (TIMP1) and collagen types II/IV secretion in 3D vessel models. The protective/inhibitory effects of adiponectin were re-analysed by inhibiting adiponectin receptor 1 (AdipoR) and AdipoR2 expression in endothelial cells using RNAi technology. In the 3D vessel models, adiponectin blocked the leptin-stimulated secretion of collagen types II/IV and TIMP1 while significantly increasing MMP2/9 activity. In endothelial cells, adiponectin induced phosphorylation of AMPK, thereby suppressing leptin-mediated STAT3 phosphorylation through induction of SOCS3 in smooth muscle cells. Our findings indicate that adiponectin disrupted the leptin-induced vascular ECM remodelling via AdipoR1 and enhanced AMPK signalling in endothelial cells, which, in turn, promoted SOCS3 up-regulation in smooth muscle cells to repress leptin-stimulated phosphorylation of STAT3.

## Introduction

While obesity has become a major public health concern, particularly in adolescents, the prevalence of hypertension has been increasing (Egan *et al*. 2010, Shay *et al*. 2013, Zhao *et al*. 2013). Accumulating evidence has shown obesity to be a major risk factor for hypertension; however, it is not always associated with hypertension (Nguyen & Lau 2012). Recent studies have shifted the paradigm of adipose tissue from a simple energy-storage organ to an active contributor to inflammatory and metabolic effects (Raucci *et al*. 2013) that are characterised by abnormal adipokine production and the activation of pro-inflammatory signalling (Fuentes *et al*. 2013). This could possibly explain the interrelationship between obesity and hypertension.

Leptin, a 16-kDa adipokine encoded by the *Ob* gene, has been examined for its role in influencing the hypothalamus and thus controlling appetite (Zhang *et al*. 1994). However, exposure to high doses of leptin has been associated with hepatic and renal fibrosis (Saxena *et al*. 2002). Fibrosis has been attributed to extracellular matrix (ECM) remodelling, which is the result of disruption in the balance between ECM synthesis and degradation. Furthermore, arterial remodelling implicated in hypertension is associated, at least in part, with vascular ECM remodelling (Lemarie *et al*. 2010). ECM remodelling is largely determined by the rate of collagen synthesis and the balance of the degradative enzymes – matrix metalloproteinases (MMPs) – with respect to a highly regulated multifunctional endogenous tissue inhibitor, the tissue inhibitor of metalloproteinases (TIMPs; Fedak *et al*. 2005). During hepatic fibrosis, leptin suppresses the expression and activity of the collagen-degrading MMPs, such as MMP2, and promotes the expression of TIMP1, an important negative regulator of MMP2 (Cao *et al*. 2007). The leptin receptor has been shown to be widely expressed in vascular cells such as umbilical vascular endothelial cells, coronary artery endothelial cells and smooth muscle cells (Kang *et al*. 2000, Knudson *et al*. 2005). However, the role of leptin in vascular ECM remodelling remains unclear.

Unlike leptin, which has profibrogenic effects, adiponectin, a 30-kDa adipokine, is anti-fibrogenic. Full-length adiponectin, which has been shown to possess biological activity (Garaulet *et al*. 2007), binds adiponectin receptor 1 (AdipoR1) (Daniele *et al*. 2012, Nigro *et al*. 2013), found primarily in the muscles, and the liver-based AdipoR2 (Yamauchi *et al*. 2003). A study by Handy *et al*. (2011) demonstrated that adiponectin knockout (*Ad* ^−/−^) mice are more susceptible to leptin-induced liver fibrosis and that recombinant adiponectin treatment could attenuate the liver fibrosis caused by leptin. Recent evidence from our laboratory has indicated that adiponectin might be a natural antagonist of leptin, and further revealed that adiponectin did not alleviate the inflammation of the blood vessels, rather it reduced leptin-induced sVACM1 secretion (Zhang *et al*. 2011).

Therefore, in view of the results described above and the paucity of literature on the opposing roles of leptin and adiponectin in fibrosis, we validated the role of leptin as a mediator of profibrogenic responses in 3D vessels. In our previous study, we had designed a 3D vessel model that mimics real blood vessels and is primarily composed of vascular endothelial cells, vascular smooth muscle cells and collagen type I (Zhang *et al*. 2011). We analysed the secretion of MMP2, MMP9, TIMP1 and collagen types II/IV in human umbilical vein endothelial cells (HUVECs) and human umbilical arterial smooth muscle cells (HUASMCs) by using the 3D vessel model. Furthermore, we evaluated the leptin-antagonist function of adiponectin in our 3D vessel model in order to elucidate the effects of receptors and the underlying signal transduction pathways of adiponectin.

## Materials and methods

### Preparation of HUVECs and HUASMCs

The present study was approved by the Ethics Committee of Shanghai Jiao Tong University, Shanghai, China. The protocol was performed according to the protocol used in our previous study (Zhang *et al*. 2011).

Immunohistochemistry and immunofluorescence studies were carried out for HUVECs and HUASMCs by using the primary antibodies against CD34 (anti-CD34; dilution, 1:100; Santa Cruz), VEGF (anti-VEGF; dilution, 1:50; Santa Cruz), factor VIII (anti-factor VIII; dilution, 1:50; Santa Cruz) and α-SMA (anti-smooth muscle α-actin; dilution, 1:200; Santa Cruz). The immunostained cells were then visualised under an inverted microscope (Olympus).

### Construction of the 3D co-culture model

**Figure 1:**
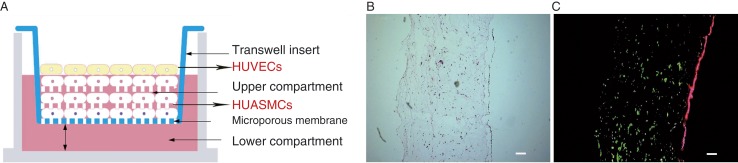
(A) Schematic illustration of the 3D co-culture model. (B) H&E-stained section of the 3D co-culture model. (C) Positive staining with FITC-conjugated mouse anti-human α-SMA (green) and positive staining with TRITC-conjugated mouse anti-human CD34 (red). Scale bars=10 μm. The results obtained were similar to those from our previous study (Zhang *et al*. 2011).

The procedure was carried out in accordance with the protocol used in our previous study (Zhang *et al*. 2011). Briefly, the HUVECs from the second, third or fourth passage and the HUASMCs from one of the passages between passage two and eight were chosen randomly and employed for the construction of the 3D co-culture model. The 3D co-culture model was composed of HUASMCs in the gel and HUVECs on the gel surface, identical to their distribution in a normal artery (Fig. 1A). This model is referred to as 3D culture because the cells grow in a support gel. The cell densities of the HUASMCs and HUVECs used were 10^6^–10^7^ and 3–5×10^5^/well respectively. A low density of HUVECs was used because larger numbers of cells tend to mass together and thus become too dense to suspend. Type I collagen (Gibco), 0.1 mM NaOH and 10× DMEM (high glucose, Gibco) were prepared with cold water. Briefly, 200 μl of type I collagen (5 mg/ml) was added to 12 μl NaOH (0.1 mM) with constant stirring, followed by the addition of 23 μl of 10× DMEM to obtain a collagen solution with the final pH near 7. Subsequently, the HUASMCs were resuspended immediately in 100 μl of the collagen solution. Then, this mixture was transferred into the upper compartment of a Transwell (3495, Corning, Chicago, IL, USA). A solid gel was obtained following incubation at 37 °C for 20 min. Finally, the HUVECs were resuspended in 200 μl of F12K (Gibco) and were subsequently transferred onto the surface of the gel. An additional 1000 μl of F12K was added to the lower compartment of the Transwell for continuous culture. The medium was renewed every 12 h during the first 2 days.

### RNA interference of *ADIPOR1* and *ADIPOR2* in HUVECs

The HUVECs were seeded at 8×10^4^ cells/well into 24-well plates in 0.5 ml of an appropriate growth medium containing serum and antibiotics. The cells were incubated under normal growth conditions (37 °C and 5% CO_2_) for a short period. Subsequently, 37.5 ng of siRNA specifically targeting *ADIPOR1* and/or *ADIPOR2* (Qiagen) was diluted in 100 μl of culture medium lacking serum to give a final siRNA concentration of 5 nM. Then, 3 μl of HiPerFect Transfection Reagent (Qiagen) was added to the diluted siRNA, and the solution was mixed by vortexing. The cells were incubated along with the transfection complexes under their normal growth conditions. Gene silencing was monitored after 72 h by testing the expression of *ADIPOR1* and *ADIPOR2* in HUVECs by using flow cytometry. Every group included a positive control, a negative control, uninfected control and mock-transfected cells.

### ELISA

The 3D model was divided into four groups: leptin (200 ng/ml; PROSPEC, East Brunswick, NJ, USA), adiponectin (10 μg/ml; PROSPEC), leptin+adiponectin and control. Then, 200 μl of the culture solution (control) or 200 μl of the culture solution containing leptin, adiponectin, or leptin+adiponectin was added into the upper compartment of the Transwell. The supernatants were collected from both the lower and the upper compartments after 24 h and then centrifuged at 1000 ***g*** for 5 min at 4 °C. Subsequently, they were analysed by ELISA to evaluate collagen types II/IV (Cosmo Bio, Toyo, Koto-ku, Tokyo, Japan), TIMP1 (R&D Systems, Minneapolis, MN, USA) and MMP2/9 levels (BioVendor, Heidelberg, Germany).

The 3D models constructed using HUVECs that were transfected with siRNA specifically targeting *ADIPOR1* or *ADIPOR2* were further divided into four groups: leptin (200 ng/ml), adiponectin (10 μg/ml), leptin+adiponectin and control. The levels of collagen types II/IV, TIMP1 and MMP2/9 were determined by using ELISA.

The 3D models treated with both leptin and adiponectin for 24 h were divided into five groups based on the different pre-treated compounds: control, compound C (CST, Danvers, MA, USA; 10 μM, 2 h), PD98059 (CST; 5 μM, 2 h), okadaic acid (CST; 0.5 μM, 1 h) and SB202190 (CST; 20 μM, 2 h). The above blockers were added before treatment. The concentration of the blockers used was chosen on the basis of results from our earlier study (Zhang *et al*. 2011). Following the treatment with the blocker, the expression of collagen types II/IV, TIMP1 and MMP2/9 was evaluated.

### Real-time RT-PCR

The HUVECs and HUASMCs, including those grown on top of the gel, were digested with 0.125% trypsin/EDTA (Gibco) centrifuged at 1500 r.p.m. for 5 min, and total RNA was extracted by using TRIzol (Invitrogen). RT was carried out with the PrimeScript RT Reagent Kit (Takara, Dalian, Shandong, China; DRR037A) by using a PTC-200 PCR instrument (Bio-Rad). The real-time RT-PCR primers for collagen types II/IV, TIMP1 and MMP2/9 were designed and provided by Takara (Table 1). The PCR was carried out with the one-step SYBR PrimeScript RT-PCR Kit II (FQ Kit, Takara DRR083A) by using a PTC-200 DNA Engine Cycler and CFD-3200 Opticon Detector (Bio-Rad).

The mRNA levels of collagen types II/IV, *TIMP1* and *MMP2/9* from the different 3D models described earlier were evaluated by using RT-PCR.

### Western blotting analysis

The cells were prepared as described for the ELISA and subsequently lysed in RIPA buffer. The total protein lysates were resolved on a 7.5% SDS–PAGE gel and blotted on a polyvinyl derivative membrane. The blots were then incubated with the primary antibodies against pAMPK/AMPK (CST, 1:1000 dilution), SOCS3 (Abcam, Cambridge, MA, USA; 1:500 dilution), and pSTAT3/STAT3 (CST, 1:1000 dilution) overnight at 4 °C. Then, the blots were incubated with goat anti-mouse IgG conjugated with HRP (CST, 1:5000 dilution) for 1 h at room temperature. The signals were detected by using ECL Reagent (GE Healthcare, Piscataway, NJ, USA). The expression of pAMPK/AMPK was analysed in HUVECs, while the expression of pSTAT3/STAT3 and SOCS3 was assessed in HUASMCs. The HUASMCs were prepared and collected as described for the ELISA and the expression of SOCS3 was evaluated.

### Statistical analyses

All data were analysed by using the SPSS 19.0 Software (SPSS, Inc.). One-way ANOVA and the least significant difference *t*-test were used to determine significant differences between the means of different groups. All data have been expressed as mean±s.d. Differences at the 95% CI (*P*<0.05) were considered significant.

## Results

### Characteristics of HUVECs and HUASMCs in 3D co-culture models

In this study, both the HUVECs and HUASMCs were positive by staining for VEGF and factor VIII. In contrast to the HUASMCs, all the HUVECs showed strongly positive CD34 staining. We found that only the HUASMCs showed α-SMA immunopositivity, in comparison with the HUVECs.

As in our previous study, the behaviour of the 3D co-culture models was identical to that of the normal vascular structure (Zhang *et al*. 2011). Interestingly, the HUVECs in the 3D co-culture model formed a structure similar to that of the vascular lumen on the gel surface. In contrast, the growth of HUASMCs in 3D culture was characterised by the formation of crosslinks between the cells, thus resulting in a 3D multilayer monoculture that was clearly distinct from the traditional single-layer culture (Zhang *et al*. 2011). Haematoxylin and eosin (H&E) and immunofluorescence staining of the 3D co-culture model showed a structure similar to that of the umbilical artery (Fig. 1B and C).

### Influence of leptin and adiponectin on the expression of collagen types II and IV, TIMP1, MMP2 and MMP9

**Figure 2:**
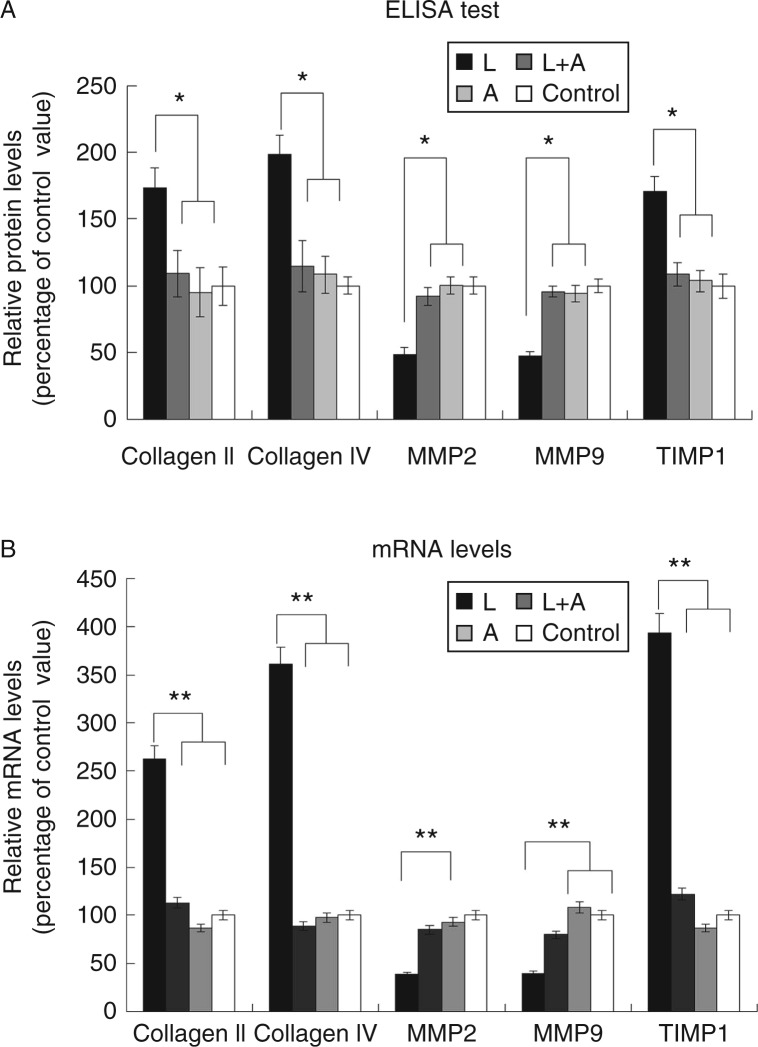
Effects of leptin and adiponectin on the protein and mRNA expression of collagen II/IV, MMP2/9 and TIMP1 in 3D vessel models. (A) When compared with the control, the relative protein levels of collagen types II and IV, MMP2, MMP9, and TIMP1 were as follows: leptin group: 1.73, 1.99, 0.49, 0.47 and 1.713 respectively; leptin+adiponectin group: 1.09, 1.15, 0.92, 0.95 and 1.09 respectively and adiponectin group: 0.95, 1.08, 1.00, 0.94 and 1.04 respectively. *n*=6. *P<0.05 (L, leptin group; A, adiponectin group and L+A, leptin+adiponectin group). (B) When compared with the control, the relative mRNA levels of collagen types II and IV, *MMP2*, *MMP9* and *TIMP1* were as follows: leptin group: 2.63, 3.61, 0.39, 0.4 and 3.94 respectively; leptin+adiponectin group: 1.13, 0.89, 0.85, 0.8 and 1.22 respectively and adiponectin group: 0.87, 0.98, 0.93, 1.086 and 0.87 respectively. *n*=6. **P<0.05 (L, leptin group; A, adiponectin group and L+A, leptin+adiponectin group).

When compared with the levels for the control, the relative protein levels of collagen types II and IV, MMP2, MMP9 and TIMP1 were as follows: leptin group: 1.73, 1.99, 0.49, 0.47 and 1.713 respectively; leptin+adiponectin group: 1.09, 1.15, 0.92, 0.95 and 1.09 respectively and adiponectin group: 0.95, 1.08, 1.00, 0.94 and 1.04 respectively. The products in the lower compartment were mainly generated by the HUASMCs, while those in the upper compartment were mainly generated by the HUVECs. In this study, the expression of collagen types II/IV and TIMP1 was found to be significantly higher in the leptin group than that in the control group (*P*<0.05). However, there was no significant difference in their expression patterns between the control group and the adiponectin or the leptin+adiponectin group. In contrast, the expression patterns of MMP2 and MMP9 among the different groups were the exact opposite of those exhibited by collagen types II and IV and TIMP1 (Fig. 2A).

The real-time PCR results were consistent with those from the ELISA (Fig. 2B). When compared with the control, the relative mRNA levels of collagen types II and IV, *MMP2*, *MMP9* and *TIMP1* were as follows: leptin group: 2.63, 3.61, 0.39, 0.4 and 3.94 respectively; leptin+adiponectin group: 1.13, 0.89, 0.85, 0.8 and 1.22 respectively and adiponectin group: 0.87, 0.98, 0.93, 1.086 and 0.87 respectively.

**Figure 3:**
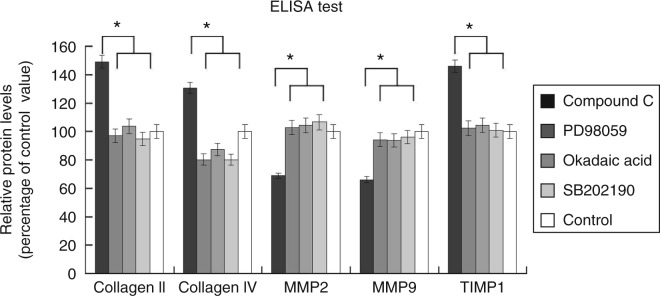
Effect of different blockers on the expression of collagen types II/IV, MMP2/9 and TIMP1 in 3D co-culture models pre-treated with leptin and adiponectin. When compared with the control group, the relative protein levels of collagen II, collagen IV, MMP2, MMP9 and TIMP1 in the compound C group were 1.49, 1.30, 0.69, 0.66 and 1.46 respectively. There were no significant differences among the other groups. *n*=6. *P<0.05.

Furthermore, we investigated the effect of different cellular signal transduction modulators on the expression of collagen types II/IV, TIMP1, MMP2 and MMP9 in the 3D models treated with both leptin and adiponectin. The different modulators used in this study were compound C, PD98059, okadaic acid and SB202190, which specifically inhibit the activation of AMPK, ERK1/2, PP2A and p38 respectively. When compared with the levels for the control group, the relative protein levels of collagen II, collagen IV, MMP2, MMP9 and TIMP1 in the compound C group were 1.49, 1.30, 0.69, 0.66 and 1.46 respectively (Fig. 3). Notably, the expression of collagen types II/IV and TIMP1 was found to be significantly higher in the compound C group than in the control group (*P*<0.01). The expression of MMP2 and MMP9 was found to be significantly lower in the compound C group than in the control group (*P*<0.01). However, there was no significant difference in their expression levels between the control group and those treated with PD98059, okadaic acid or SB202190 (Fig. 3).

### Gene silencing of *ADIPOR1* and *ADIPOR2* in the HUVECs

**Figure 4:**
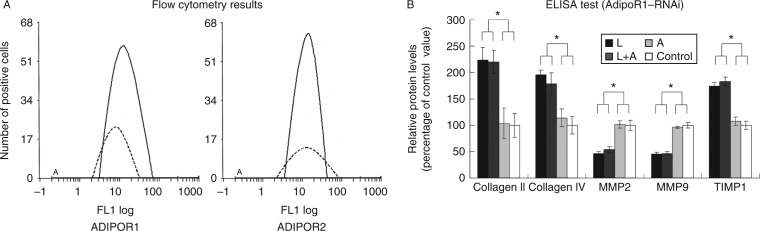
Effects of gene silencing in HUVECs. (A) The inhibition rates of ADIPOR1 (left) and ADIPOR2 (right) for HUVECs, determined using flow cytometry, were 71.83±1.45 and 74.89±1.12% respectively. However, no significant difference was observed in the inhibition rates of ADIPOR2 (2.76±0.54%; when ADIPOR1 was targeted) and ADIPOR1 (3.72±0.42%; when AdipoR2 was targeted) when compared with the control. (B) When compared with the control, the relative protein levels of collagen II, collagen IV, MMP2, MMP9 and TIMP1 were as follows: leptin group: 2.24, 1.96, 0.46, 0.46 and 1.74 respectively; leptin+adiponectin group: 2.12, 1.78, 0.54, 0.47 and 1.83 respectively and adiponectin group: 1.04, 1.14, 1.02, 0.96 and 1.08 respectively. *n*=6. *P<0.05 (L, leptin group; A, adiponectin group and L+A, leptin+adiponectin group).

To evaluate the role of the AdipoRs in the HUVECs, species-specific phosphorothioate-modified antisense oligonucleotides were designed. The transfection of siRNAs specifically targeting human *ADIPOR1* and *ADIPOR2* into HUVECs significantly reduced the protein expression levels of ADIPOR1 and ADIPOR2 (Fig. 4A). Moreover, it was observed that the siRNAs did not cross-react with targets. The negative control siRNA sequence had no effect on the protein expression of ADIPOR1 or ADIPOR2.

The inhibition rates of *ADIPOR1* and *ADIPOR2* for HUVECs, determined using flow cytometry, were 71.83±1.45 and 74.89±1.12% respectively. However, no significant difference was observed in the inhibition rates of *ADIPOR2* (2.76±0.54% when *ADIPOR1* was targeted) and *ADIPOR1* (3.72±0.42% when *ADIPOR2* was targeted) in comparison with the control.

In comparison with the control, the relative protein levels of collagen II, collagen IV, MMP2, MMP9 and TIMP1 were as follows: leptin group: 2.24, 1.96, 0.46, 0.46 and 1.74 respectively; leptin+adiponectin group: 2.12, 1.78, 0.54, 0.47 and 1.83 respectively and adiponectin group: 1.04, 1.14, 1.02, 0.96 and 1.08 respectively. Therefore, knockdown of *ADIPOR1* from HUVECs in the 3D co-culture models resulted in a significant increase in the expression levels of collagen types II/IV and TIMP1 in the leptin and leptin+adiponectin groups as compared with those in the control group (*P*<0.01), whereas their expression levels in the adiponectin group did not significantly differ from those in the control group (*P*>0.01). There was no significant difference in the knockdown efficiency of *ADIPOR1* between the adiponectin and the control groups.

In the 3D co-culture models with *ADIPOR2*-knocked down HUVECs, there was a significant increase in the expression levels of collagen types II/IV and TIMP1 in the leptin group as compared with those for the control group (*P*<0.01; data not shown).

However, the expression patterns of MMP2 and MMP9 in the *ADIPOR1*- and *ADIPOR2*-knocked down HUVECs displayed the opposite trend when compared with those of collagen types II/IV and TIMP1 (Fig. 4B).

### Influence of leptin and adiponectin on the expression of pAMPK/AMPK in HUVECs and of pSTAT3/STAT3 and SOCS3 in HUASMCs

**Figure 5:**
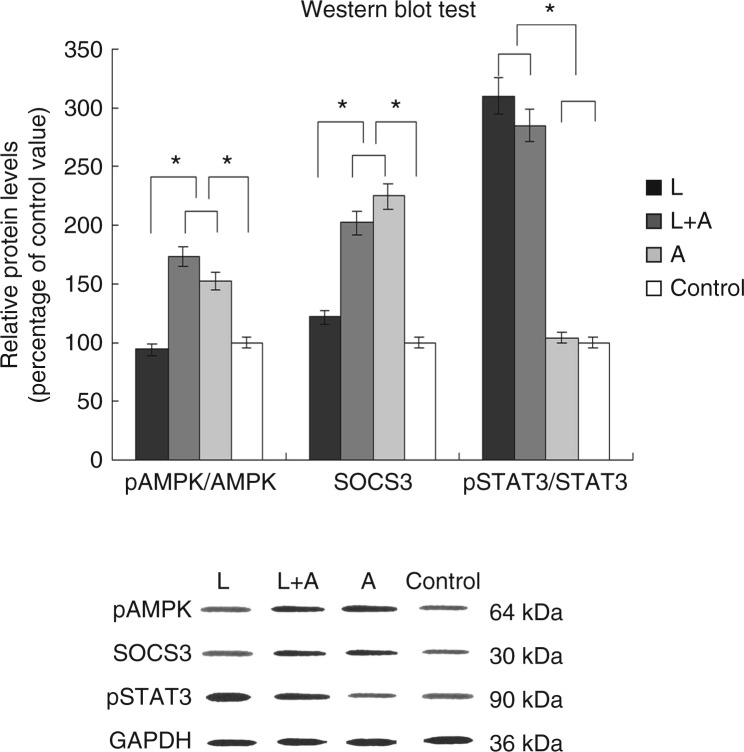
Effects of leptin and adiponectin on the protein levels of pAMPK, SOCS3 and pSTAT3 in 3D models. When compared with the control, the relative protein levels of pAMPK, SOCS3 and pSTAT3 were as follows: leptin group: 0.94, 1.22 and 3.10 respectively; leptin+adiponectin group: 1.74, 2.02 and 2.85 respectively and adiponectin group: 1.52, 2.24 and 1.04 respectively. *n*=6. *P<0.05 (L, leptin group; A, adiponectin group and L+A, leptin+adiponectin group).

In comparison with the control, the relative protein levels of pAMPK, SOCS3 and pSTAT3 were as follows: leptin group: 0.94, 1.22 and 3.10 respectively; leptin+adiponectin group: 1.74, 2.02 and 2.85 respectively and adiponectin group: 1.52, 2.24 and 1.04 respectively. In this study, the expression level of pAMPK in HUVECs in the adiponectin group was significantly higher than that in the control group (*P*<0.01; Fig. 5).

Notably, the levels of pSTAT3 expressed by HUASMCs in the leptin and the leptin+adiponectin groups were found to be significantly higher (*P*<0.01) than those of the control group. However, no significant difference was seen between the adiponectin and the control groups. In contrast, the expression levels of SOCS3 in HUASMCs from the adiponectin group were significantly higher than those for the control group (Fig. 5).

**Figure 6:**
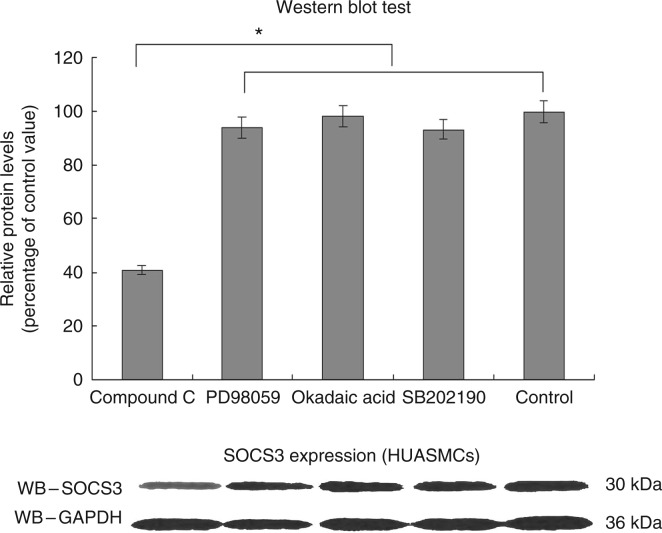
Effects of different blockers on the expression of SOCS3 in 3D models pre-treated with leptin and adiponectin. When compared with the control, the relative protein levels of SOCS3 in the compound C group, PD98059 group, okadaic acid group and SB202190 group were 0.41, 0.94, 0.98 and 0.93 respectively. *n*=6. *P<0.05.

When compared with the results for the control, the relative protein levels of SOCS3 in the compound C group, PD98059 group, okadaic acid group and SB202190 group were 0.41, 0.94, 0.98 and 0.93 respectively. Surprisingly, the levels of SOCS3 expressed by HUASMCs in the compound C group were found to be significantly lower in comparison with those for the other groups (*P*<0.01; Fig. 6).

## Discussion

In the present study, we employed a 3D co-culture model that closely mimicked a normal arterial vessel when compared with the traditional cell culture systems (Zhang *et al*. 2011). One of the primary advantages of employing our established 3D model in this study was the ability to analyse the interaction between endothelial cells and smooth muscle cells, which is difficult to achieve with traditional 2D cell culture systems, which that pose major technical challenges. In this study, we did not compare the findings obtained using our 3D co-culture model with that of a traditional 2D cell culture system because the feasibility and versatility of our established 3D models over the commonly employed 2D cell culture systems have already been demonstrated and proven in our previous study (Zhang *et al*. 2011).

Both obesity and hypertension have been categorised as chronic, low-grade inflammatory diseases (Dhande *et al*. 2013). It is well known that vascular ECM remodelling leads to arteriosclerosis and, subsequently, hypertension (Lemarie *et al*. 2010). Convincing evidence from numerous studies indicates a dynamic role of adipokines in hypertension. Although the primary functions of the adipokines adiponectin and leptin are related to metabolism and satiety, several key studies have demonstrated their involvement in ECM homeostasis (Schram *et al*. 2010, Zibadi *et al*. 2011).

In human plasma, adiponectin circulates as a trimer, a hexamer and a high-molecular-weight multimer. For the present study, we used human total adiponectin/adipocyte complement-related protein 30 (ACRP30), which is an adipocyte-derived protein with wide-ranging paracrine and endocrine effects on metabolism and inflammation. Moreover, in our previous study, we found human total adiponectin to be more active than other forms.

In this study, we assessed the changes in the expression levels of collagen types II/IV, MMP2, MMP9 and TIMP1, which are known to be involved in ECM remodelling, by using ELISA and further validated the results obtained by using real-time RT-PCR (Zibadi *et al*. 2011). First, we demonstrated that leptin could promote the synthesis of collagen. Leptin pre-treatment in 3D vessel models resulted in the down-regulation of *MMP2* and *MMP9*, which are involved in collagen degradation, while simultaneously enhancing the *TIMP1* activity and increasing the expression of collagen types II/IV. Therefore, our results demonstrate the predominance of collagen synthesis over degradation in the presence of leptin. Our findings are consistent with results from a previous study by Saxena *et al*. (2002), showing that CCl4-treated leptin-deficient (*ob/ob*) mice failed to develop liver fibrosis. Taken together, these data indicate that leptin contributes to vascular ECM remodelling, which is one of the novel findings of this study.

While leptin is a profibrogenic adipokine, multiple studies have indicated that adiponectin is anti-fibrogenic, although the underlying mechanisms have not been elucidated yet (Bertolani & Marra 2008). Hence, we investigated whether adiponectin could antagonise the effects of leptin. To achieve this, we employed 3D vessel models with the combined presence of leptin and adiponectin. Notably, adiponectin elevated the levels of *MMP2* and *MMP9*, which were diminished by leptin, and reduced *TIMP1* activity while down-regulating the expression of collagen types II/IV. Interestingly, their expression patterns remained unchanged in the presence of adiponectin alone. Therefore, based on these findings, we propose that adiponectin displays its antifibrogenic effects only in the presence of leptin and that pre-treatment with leptin is necessary in order to study the antagonistic actions of adiponectin.

To our knowledge, the present study is the first to comprehensively explore the potential role of adiponectin and its receptors in vascular ECM remodelling by using 3D co-culture models. Using a pair of *ADIPOR1*- and *ADIPOR2*-specific antisense oligonucleotides, we evaluated the role of each receptor in the anti-leptin actions of adiponectin. Notably, the knockdown of *ADIPOR1* from HUVECs in the 3D co-culture models resulted in a significant increase in the expression levels of collagen types II and IV and TIMP1 in the leptin+adiponectin group, compared with those in the control group (*P*<0.01). In contrast, there was no significant difference in the knockdown efficiency of *ADIPOR2* between the control and the leptin+adiponectin groups. Taken together, these findings indicate that only the inhibition of *ADIPOR1* was capable of reversing the anti-leptin actions of adiponectin.

Interestingly, in this study, there was a significant increase in the levels of pSTAT3 expressed by HUASMCs in the leptin and the leptin+adiponectin groups (*P*<0.01) when compared with that of the control group, indicating that leptin promotes the phosphorylation of STAT3 in 3D vessel models. However, there was no significant difference between the pSTAT3 levels for the adiponectin and the control groups (Fig. 5). Collectively, these results indicate that adiponectin suppressed leptin-stimulated phosphorylation of STAT3 in the leptin+adiponectin group, thus indicating that the inhibitory effects of adiponectin in ECM remodelling rely on the leptin-stimulated phosphorylation of STAT3.

Furthermore, we examined the role of different cellular signal transduction modulators, namely compound C, PD98059, okadaic acid and SB202190, which could specifically inhibit the activation of AMPK, ERK1/2, PP2A and p38, respectively, in 3D vessel models (with both leptin and adiponectin), and investigated their effect on the expression of collagen types II and IV, TIMP1, MMP2 and MMP9. Strikingly, we observed that in the presence of compound C, a specific inhibitor of AMPK, the antagonistic effect of adiponectin was completely abolished (Fig. 3), while the addition of other blockers had little influence on it. In addition, using western blotting analysis, we observed that there was an increase in the expression levels of pAMPK in HUVECs and SOCS3 in the HUASMCs of the adiponectin group (Fig. 5), thus lending support to our finding that the inhibitory effects of adiponectin on leptin are mediated by the activation of the AMPK signal transduction pathway. Therefore, this implies that adiponectin enhanced the activation of AMPK signalling (Fig. 5) and SOCS3 protein levels either alone or in the presence of leptin. Our results are corroborated by results from a previous study, which indicated that SOCS3 negatively regulates leptin signalling (Handy *et al*. 2010). Furthermore, collectively, these findings provide a plausible molecular explanation for why adiponectin exerted no influence on the expression of *MMP2*, *MMP9*, *TIMP1* and collagen types II/IV in the absence of leptin (Fig. 2).

In order to confirm the relationship between the activation of AMPK signalling and the corresponding increase in SOCS3 levels, we assessed the influence of different signal transduction modulators on SOCS3 expression by using the western blotting technique (Fig. 6). Surprisingly, the levels of SOCS3 expressed by HUASMCs declined significantly only in the presence of compound C (*P*<0.01; Fig. 6).

Taken together, based on our findings, we speculate that adiponectin reversed the actions of leptin by increasing the expression of SOCS3 in HUASMCs through the activation of the AMPK signal transduction pathway via ADIPOR1 in HUVECs. Our study elucidated a novel plausible mechanism underlying the molecular events involved in adiponectin-mediated AMPK signal transduction. It is interesting to note that we obtained these novel findings because we chose to use both HUVECs and HUASMCs together, in our established 3D co-culture models, unlike several previous studies, which employed different cell types separately. Therefore, our study highlights the molecular mechanisms of the crosstalk between HUVECs and HUASMCs. Future studies that elucidate the mechanism of this crosstalk are warranted.

It is critically important to investigate the potential of obesity-induced vascular ECM remodelling for targeting molecular therapy. In our study, leptin appeared to be responsible for vascular ECM remodelling. As expected, adiponectin exerted inhibitory effects on leptin-induced effects. However, at present, we cannot infer whether SOCS3 is the only pathway involved in adiponectin down-regulation of leptin signalling. Moreover, *in vivo* experiments using *Ob/Ob* mice and *Ad* ^−/−^ mice are required to validate the fibrosis-opposing effects of adiponectin and further corroborate the mechanisms indicated by the *in vitro* experiments. Future studies investigating the role of calreticulin or T-cadherin receptors, which also function as AdipoRs in addition to ADIPOR1 or ADIPOR2, are warranted (Hug *et al*. 2004, Takemura *et al*. 2007, Parker-Duffen *et al*. 2013). However, it should be mentioned that the hypertension-inducing effect of leptin could be attributed to additional factors other than fibrosis, such as angioneogenesis, increased sympathetic tone and smooth muscle cell hyperplasia (Eikelis *et al*. 2003, Shan *et al*. 2008, Chai *et al*. 2014).

In conclusion, our study demonstrated that adiponectin inhibited the leptin-induced vascular ECM remodelling via AdipoR1 and enhanced AMPK signalling in HUVECs, which, in turn, promoted SOCS3 up-regulation in HUASMCs to repress the leptin-stimulated phosphorylation of STAT3.

## Figures and Tables

**Table 1:** The real-time RT-PCR primers for collagen types II/IV, TIMP1 and MMP2/9

**Gene name**	**GenBank accession number**	**Forward**	**Reverse**
Collagen II	NM_033150	CAATAGCAGGTTCACGTACAC	TCGATAACAGTCTTGCCCCA
Collagen IV	NM_001845	CTCTACGTGCAAGGCAATGA	AGAACAGGAAGGGCATTGTG
MMP2	NM_004530	CAACTACAACTTCTTCCCTCGCA	GGTCACATCGCTCCAGACTTG
MMP9	NM_004994	GCATAAGGACGACGTGAATGGC	CGGTGTGGTGGTGGTTGGAG
TIMP1	NM_003254	AAGGCTCTGAAAAGGGCTTC	GAAAGATGGGAGTGGGAACA
GAPDH	NM_002046	TGCACCACCAACTGCTTAGC	GGCATGGACTGTGGTCATGAG
